# Sanitization Efficacy of Slightly Acidic Electrolyzed Water against pure cultures of *Escherichia coli, Salmonella enterica, Typhimurium*, *Staphylococcus aureus* and *Bacillus cereus spores*, in Comparison with Different Water Hardness

**DOI:** 10.1038/s41598-019-40846-6

**Published:** 2019-03-13

**Authors:** Hyun-Ji Kim, Charles Nkufi Tango, Ramachandran Chelliah, Deog-Hwan Oh

**Affiliations:** 1 Department of Food Science and Biotechnology, School of Bioconvergence Science and Technology, Kangwon National University, Chuncheon, Gangwon 24341 Republic of Korea; 2 Division of Cancer Epidemiology and Management, Center for Uterine Cancer, National Cancer Center, Ilsandong-gu, Goyang Republic of Korea

## Abstract

The Influence of water source on the production of slightly acidic electrolyzed water (SAEW) and its sanitization efficacy were investigated. Two different water sources (tap water (TW) and underground water (UGW)) were applied to produce slightly acidic electrolyzed water (SAEW) at same setting current, with similar electrolyte flow rate (EFR) and concentration. Properties of SAEW were evaluated based on pH, Available chlorine concentration (ACC) and oxidation-reduction potential (ORP). Methods for the optimization of SAEW production process was examined to obtain high ACC value by implanting different types of electrolytes. Effect of ACC and pH of SAEW were evaluated *in vitro* towards inactivate foodborne pathogens. The results indicated that TW with hardness of 29 ppm produced effectively SAEW than through UGW (12 ppm) using electrolytes. Likewise, low water hardness could be reinforced by combining HCL with a salt (NaCl or KCL). The optimized SAEW production system was determined at 4% HCl + 2.0 M KCL with EFR of 2 mL/min and 4% HCl + 3.0 M KCL with EFR of 2 mL/min resulting in higher ACC value of 56.5 and 65.5 ppm, respectively using TW. Pathogenic vegetative cells were completely inactivated within 1 min of treatment in SAEW with 20 ppm. Viability observations using Confocal and TEM Microscopy, Flow cytometry, and antimicrobial activity were carried out to confirm the sanitizing effect and cell membrane disruption. Based on the experimental results obtained, it provides a foundation for future advancement towards commercial application of SAEW in the food and agricultural industries.

## Introduction

Detrimental effects of traditional chlorine treatment have been reported, including corrosion to surfaces and negative effects on human health and the environment. Recent studies have highlighted that slightly acidic electrolyzed water (SAEW) is one potential alternative to traditional chlorine treatment^1,2^. SAEW is generated by electrolysis of hydrochloric acid (HCL) and/or soft salt solutions using an electrolytic cell without membrane between anode and cathode.

**Figure 1:**
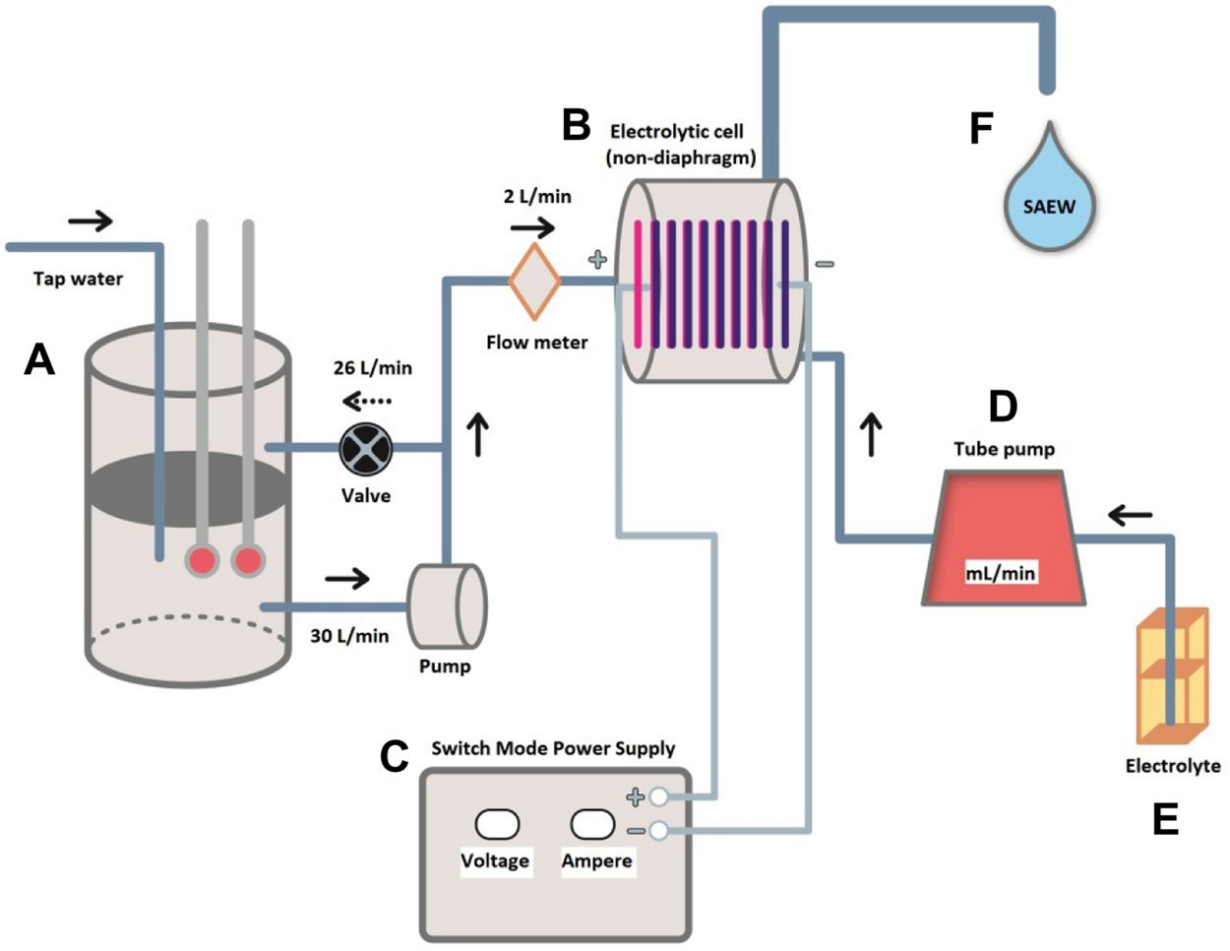
Schematic diagram of the electrolyzed water generator system implemented for the production of SAEW in the study. **(A)** water tank, **(B)** electrolytic cell, **(C)** power supply, **(D)** master flex, **(E)** electrolyte, **(F)** SAEW produced. *Process description: Tap water flows into the water tank, **(A)** and undergoes water electrolysis in the electrolytic cell, **(B)**. Amperage and voltage have been arranged through switch mode power supply, **(C)** and electrolyte, **(E)** is controlled by master flex, **(D)** that drives flow rate of electrolyte. Finally, SAEW, **(F)** is generated through the EW generator system.

SAEW with a pH range of 5.0–6.5, has advantages of possessing high antimicrobial efficacy due to high amount of hypochlorous acid (HOCl) and reduction of corrosion in food industry plants and less damage to human health and environment^3,4^. A schematic mechanism illustration of the slightly electrolyzed water generator system is represented in Fig. 1 and Supplement 1 and 2.

The major reaction equations are expressed as below:

$$\begin{array}{rcl}{\rm{Anode}}:(1)\,2{{\rm{Cl}}}^{-} & \to  & {\bf{C}}{{\bf{l}}}_{{\bf{2}}}(\,\uparrow \,)+2{{\rm{e}}}^{-}\\ {\rm{Cathode}}:(2)\,2{{\rm{H}}}^{+}+2{\rm{e}}\, & \to  & {{\rm{H}}}_{2}(3){{\rm{Na}}}^{+}+{{\rm{OH}}}^{-}\to {\bf{N}}{\bf{a}}{\bf{O}}{\bf{H}}\\ {{\rm{Cl}}}_{2}+{{\rm{H}}}_{2}{\rm{O}} & \to  & {\bf{H}}{\bf{O}}{\bf{C}}{\bf{l}}+{{\rm{H}}}^{+}+{{\rm{Cl}}}^{-}\\ {{\rm{Cl}}}_{2}+2{{\rm{OH}}}^{-} & \to  & {\bf{C}}{\bf{l}}{{\bf{O}}}^{-}+{{\rm{Cl}}}^{-}+{{\rm{H}}}_{2}\\ {{\rm{Cl}}}_{2}+2{{\rm{OH}}}^{-} & \to  & {\bf{C}}{\bf{l}}{{\bf{O}}}^{-}+{{\rm{Cl}}}^{-}+{{\rm{H}}}_{2}{\rm{O}}\\ {{\rm{Cl}}}_{2}+2{\rm{NaOH}} & \to  & 2{\bf{N}}{\bf{a}}{\bf{O}}{\bf{C}}{\bf{l}}+{\rm{NaCl}}+{{\rm{H}}}_{2}{\rm{O}}\\ {{\rm{Cl}}}_{2}+2{\rm{NaOH}} & \to  & {\bf{H}}{\bf{O}}{\bf{C}}{\bf{l}}+{\rm{NaCl}}\end{array}$$

It has been reported that flow rate, concentration and type of electrolyte, and water source affect significantly on the properties of EW. So far only few reports have been documented that the characteristics of EW and its sanitization efficiency can vary due to the hardness and pH of Supplement 3 the starting water^5^.

The U.S. Geological Survey classified that the water hardness is classified into four categories: soft water (0–60 mg/L CaCO_3_), moderately hard (60–120 mg/L CaCO_3_), and hard (120–180 mg/L CaCO_3_), and very hard (more than 180 mg/L CaCO_3_)^6^. Forghani *et al*.^7^ demonstrated that waters (TW) from two different places showed a difference in water hardness, therefore, affecting on the properties and sanitization efficacy of SAEW^7^.

Underground water (UW) contains naturally high amount of magnesium (Mg^+2^), calcium (Ca^+2^), and few other ions due to the constant contact between water and minerals in ground^8^. The presence of these minerals may influence water hardness and the quality of SAEW. There is a great chance of having slightly acidic property using underground water which may contains high amount of minerals. Moreover, it has been reported that increase in water hardness tended to increase free chlorine and sanitization efficacy of EW^5^. Therefore, the objective of this study was to evaluate the influence of water source on the different properties of SAEW and investigate the sanitizing efficacy against foodborne pathogens. Moreover, approaches for the optimization of SAEW production process to obtain high ACC value was also examined by adding different types of electrolytes. Cell viability assays were also assessed to demonstrate the cellular damage and the bactericidal efficacy of SAEW.

## Results

### Effect of water hardness on EW physicochemical properties

Properties (hardness, Ca, Mg concentration, and pH) of starting water used to produce EW are shown in Table 1A. Considering US geological survey report^9^, the both TW and UW were categorized as soft water. Water hardness is primarily the amount of calcium and magnesium, and as a lesser extent, iron in water. Groundwater tends to contain harder water hardness than tap water and can vary greater than 1000 mg/L by natural weathering of limestone, sedimentary rock, and calcium bearing minerals^7^. However, as followed by our observations, TW showed harder water hardness than UW.

**Table 1:** Properties of different waters and Electrolyzed water (EW) production conditions.

Water sample	pH	Water hardness (ppm)	Ca^2+^ (mg/l)	Mg^2+^ (mg/l)		
**(A)**						
Tap water	6.86	29	8.70	1.70		
Underground water	6.97	12	3.65	0.63		
**(B)**						
**Water sample**	**Input**			**Output**		
	**Electrolyte Flow rate (mL/min)**	**Amperage (A)**	**Voltage (V)**	**pH**	**ACC (ppm)**	**ORP (mV)**
Tap water	4.00	8.0	2.9	3.10	57	969/998
	3.00		2.3	3.23	44	952/992
	2.00		2.5	3.31	33	913/982
	1.00		3.9	4.16	16.5	818/925
Underground water	4.00	8.0	2.1	2.83	49	1088/1091
	3.00		2.4	2.86	35	905/1007
	2.00		2.5	3.03	30	937/1021
	1.00		2.8	3.65	14.5	992/995

**(A)** Basic properties of different waters used in EW production. **(B)** EW properties produced from Tap water and Underground water using various electrolyte flow rate, amperage, and fixed electrolyte concentration (6% HCl). ACC; available chlorine concentration (ppm), ORP; oxidation-reduction potential (mV).

Comparison of properties of EW produced from TW and UW using different electrolyte flow rate (EFR), fixed electrolyte concentration (6% HCL) and current were presented in Table 1B. The results showed that increase in EFR results in decreasing of pH, while increasing in ACC and ORP for the both TW and UW used in this study. Although the ACC increased in the both waters, TW appeared to produce higher ACC than UW.

### Optimization of SAEW production

The different combinations including HCL and KCL at different concentrations and flow rates to optimize the production process for SAEW were shown in Table 2. These combinations led to various EW production, including strongly acidic, acidic, and slightly acidic EWs. When increasing KCL concentration with rising electrolyte flow rate, higher ACC value was gained and there was decrease in pH for all combinations. Except for 1.0 M KCL (electrolyte concentration), SAEW with proper pH (5.0–6.5) was produced at the EFR between 1 and 2 mL/min for TW. However, using UW, SAEW was obtained with EFR at 1 mL/min for all combinations performed.

**Table 2:** Optimization of Tap and Underground Water Slightly Acidic Electrolyzed Water production system by combining 1.0–3.0 M KCl with 4% HCl and its physicochemical properties.

Water source	Input				Output		
	Electrolyte concentration	Electrolyte Flow rate (mL/min)	Amperage (A)	Voltage (V)	pH	ACC (ppm)	ORP (mV)
**Tap Water**	4% HCl (control)	4.00	12.0	3.0	3.21	42.5	1060/1074
		3.00		3.4	3.31	32	1018/1039
		2.00		3.6	3.64	31	848/974
		1.00		3.8	4.31	14.5	819/922
	4% HCl + 1.0 M KCl	4.00	12.0	3.8	3.76	64	998/1058
		3.00		4.1	3.98	53	960/1027
		2.00		4.3	4.49	47	867/923
		1.00		5.2	6.07	25	716/831
	4% HCl + 2.0 M KCl	4.00	12.0	3.2	3.47	70	901/1081
		3.00		3.3	3.92	62	968/1038
		2.00		3.5	5.18	56.5	820/943
		1.00		3.6	5.34	34	783/896
	4% HCl + 3.0 M KCl	4.00	12.0	3.0	3.56	72	916/1076
		3.00		3.1	3.92	67	951/1028
		2.00		3.2	5.66	65.5	800/889
		1.00		3.6	6.49	39	785/873
**Underground Water**	4% HCl (control)	4.00	12.0	2.7	2.96	45.5	1041/1082
		3.00		3.1	3.50	38	942/1039
		2.00		3.9	3.80	20.5	871/994
		1.00		5.2	5.43	11.5	792/871
	4% HCl + 1.0 M KCl	4.00	12.0	2.5	3.27	55	1013/1104
		3.00		3.2	3.34	54	957/1063
		2.00		4.0	4.34	42	878/1001
		1.00		4.8	5.89	20	739/858
	4% HCl + 2.0 M KCl	4.00	12.0	3.5	3.28	63	975/1064
		3.00		3.4	3.61	59	926/1029
		2.00		3.4	4.13	40	838/1023
		1.00		4.0	5.65	36	792/869
	4% HCl + 3.0 M KCl	4.00	12.0	3.0	3.18	69	951/1050
		3.00		3.1	3.44	67	948/1101
		2.00		3.1	3.71	50	934/1019
		1.00		3.3	5.93	38	769/854

ACC; available chlorine concentration (ppm), ORP; oxidation-reduction potential (mV).

Regarding these results, the optimal condition using TW to produce SAEW was observed in combination of 4% HCl + 2.0 M KCL (with EFR of 2 mL/min) and 4% HCl + 3.0 M KCL (with EFR of 2 mL/min), resulting in higher ACC value of 56.5 and 65.5 ppm, respectively. For UW, the optimal condition was found in the combination of 4% HCl + 3.0 M KCL (with EFR of 1 mL/min) resulting in higher ACC value of 38 ppm.

Diffèrent fusion, including HCL and NaCl at different concentrations and flow rates to optimise the production process for SAEW was shown in Table 3. As well as observed in KCL, increase in NaCl concentration and EFR resulted in increase of ACC value and reduction of pH value. However, SAEW was found at lower flow rate (1 mL/min) for all combination performed using the both TW and UW. The optimal condition was found to be 3 M NaCl combined (with EFR of 1 mL/min) and 2 M NaCl (with EFR of 1 mL/min), respectively for TW and UW.

**Table 3:** Optimization of Tap and underground water Slightly Acidic Electrolyzed Water production system by combining 1.0–3.0 M NaCl with 6% HCl and its physicochemical properties.

Water source	Input				Output		
	Electrolyte concentration	Electrolyte Flow rate (mL/min)	Amperage (A)	Voltage (V)	pH	ACC (ppm)	ORP (mV)
**Tap Water**	6% HCl (Control)	4.00	12.0	2.5	2.92	58	1041/1081
		3.00		2.8	3.04	46	919/1052
		2.00		3.2	3.33	34	911/1034
		1.00		4.6	4.53	17.5	847/961
	6% HCl + 1.0 M NaCl	4.00	12.0	3.3	2.96	79	1007/1123
		3.00		3.6	3.25	67	1031/1088
		2.00		4.1	4.76	43.5	912/1032
		1.00		4.7	6.33	24	755/857
	6% HCl + 2.0 M NaCl	4.00	12.0	3.3	2.92	79	1032/1115
		3.00		3.5	3.19	68.5	985/1095
		2.00		3.7	3.83	48	914/1045
		1.00		4.0	5.35	36.5	835/971
	6% HCl + 3.0 M NaCl	4.00	12.0	3.2	2.99	84	1011/1142
		3.00		3.3	3.20	71.5	959/1086
		2.00		3.4	3.66	62	929/1056
		1.00		3.7	5.82	40.5	897/915
**Underground Water**	6% HCl (Control)	4.00	12.0	3.0	2.95	50	994/1089
		3.00		3.2	3.23	36	956/1055
		2.00		3.2	3.38	29	929/1037
		1.00		4.3	4.16	15.5	841/945
	6% HCl + 1.0 M NaCl	4.00	12.0	3.1	2.92	65.5	986/1086
		3.00		3.6	3.07	60	980/1061
		2.00		3.8	4.42	52.5	970/1054
		1.00		4.3	5.24	23	808/863
	6% HCl + 2.0 M NaCl	4.00	12.0	3.1	2.94	72	1020/1125
		3.00		3.6	3.12	69	983/1089
		2.00		4.1	4.33	61	951/1009
		1.00		4.2	5.18	33.5	885/981
	6% HCl + 3.0 M NaCl	4.00	12.0	3.2	2.94	80	1011/1125
		3.00		3.4	3.10	77	985/1093
		2.00		3.4	4.27	68.5	966/1089
		1.00		3.7	5.17	33.5	844/936

ACC; available chlorine concentration (ppm), ORP; oxidation-reduction potential (mV).

This concentration was selected based on our previous study^7^. A low concentration (4%) of HCL was combined with KCL because it was less soluble in 6% HCL. These results confirm that the observations reported previously that the major factors influencing significantly on the properties of EW are salt concentration, flow rate, and current^10^.

### Effect of chlorine concentration and pH with dipping times on sanitization efficacy of SAEW against foodborne pathogens and *B. cereus* spores

**Figure 2:**
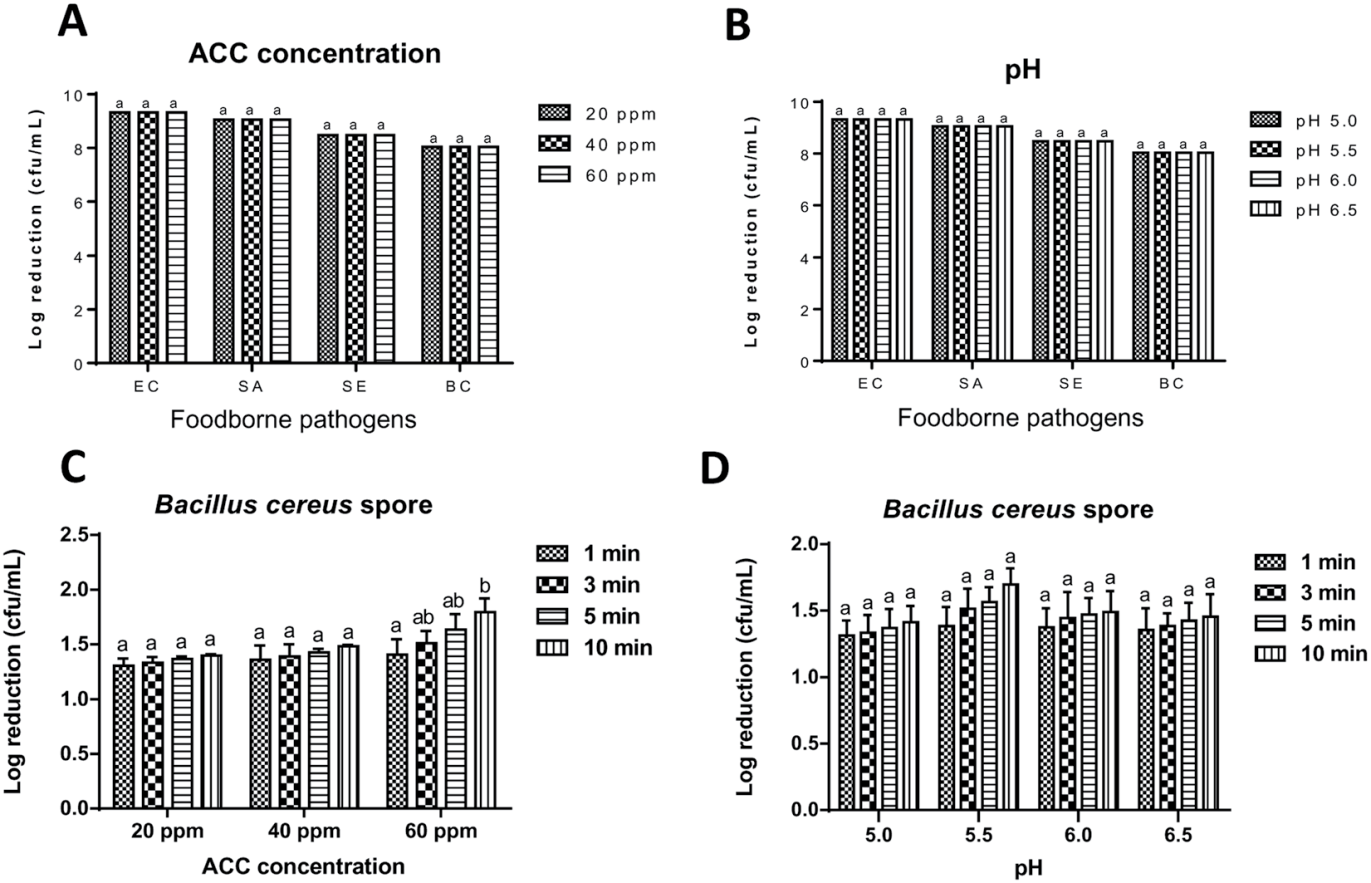
Effect of SAEW concentrations **(A)** and pH **(B)** on the inactivation of different foodborne pathogens treated at 23 ± 0.2 °C for 1 min. Effect of SAEW concentrations **(C)** and different pH **(D)** in combination with dipping times on SAEW sanitization efficacy against *B. cereus* spores at 23 ± 0.2 °C. Bars labeled with different letters in the pathogen are significantly (p > 0.05) different. The initial population of EC (*E. coli* O157:H7), SA (*S. aureus*), SE (*S. enterica*), BC (*B. cereus*), and BS (*B. cereus* spores) were 9.32, 9.06, 8.48, 8.03, and 7.65 log cfu/mL, respectively. ACC; available chlorine concentration (ppm), dipping times; 1, 3, 5, and 10 mins. ^a^More sensitive, ^b^moderate sensitive, ^c^less sensitive.

The effect of ACC on SAEW sanitization efficacy against foodborne pathogens treated at 23 ± 0.2 °C for 1 min of dipping time was represented in Fig. 2A. In order to maintain the same condition, pH was adjusted to pH 6.0 for all treatment solutions. The results indicated that the bacterial populations were completely inactivated after SAEW treatment for 1 min. This result demonstrated that at pH value of 6.0 and a free chlorine concentration of 20 ppm, SAEW treatment for 1 min, is efficient to kill approximately 8–9 Log CFU/mL of all foodborne pathogens used in the present study.

The effect of pH ranges on SAEW sanitization efficacy against foodborne pathogens treated at 23 ± 0.2 °C for 1 min of dipping time was shown in Fig. 2B. The effect of different pH was examined at the ACC of 20 ppm. The results showed that all bacterial cells were found below the detection limit (1 Log CFU/mL) for all foodborne pathogens used in the present study. The results indicated that difference in pH did not affect significantly (p > 0.05) on the chlorine concentration of 20 ppm to inactivate all bacterial cells present during 1 min of dipping treatment. This experiment confirms that in pH range of 5.0–6.5, the chlorine remains under HOCl form which is the leading factor responsible for the sanitizing effect in SAEW.

The combined effect of free chlorine concentration with dipping time on SAEW sanitization efficacy against *B. cereus* spores was presented in Fig. 2C. The treatment was performed using SAEW solution with pH value of 6.0. When increasing the contact time (from 1 to 10 min) between *B. cereus* spores and SAEW solutions with free chlorine concentration of 20 and 40 ppm, bacterial inactivation did not increase significantly (p > 0.05). However, when ACC was increased to 60 ppm, the statistical analysis showed that increasing the treatment time from 1 to 10 min caused *B. cereus* spores to decrease significantly (p < 0.05). The highest reduction of 1.80 Log CFU/mL was observed when B. cereus spores were treated with SAEW (60 ppm) for 10 min.

The effect of pH ranges combined with dipping time towards the SAEW sanitization efficacy against *B. cereus* spores was shown in Fig. 2D. To maintain the same condition for all SAEWs, ACC adjustment was implemented to 20 ppm. The bacterial reduction resulted from pH 5.0 ranged approximately 1.31, 1.33, 1.37, and 1.41 log CFU/mL for 1, 3, 5, and 10 min, respectively. The similar trends were also found to those treated with SAEW with pH value of 5.5, 6.0, and 6.5. The treatment increasing from 1 to 10 min did not affect (p > 0.05) to *B. cereus* spore inactivation for all pH ranges of SAEW used in this study.

### Confocal Laser Scanning Microscopy Analysis

**Figure 3:**
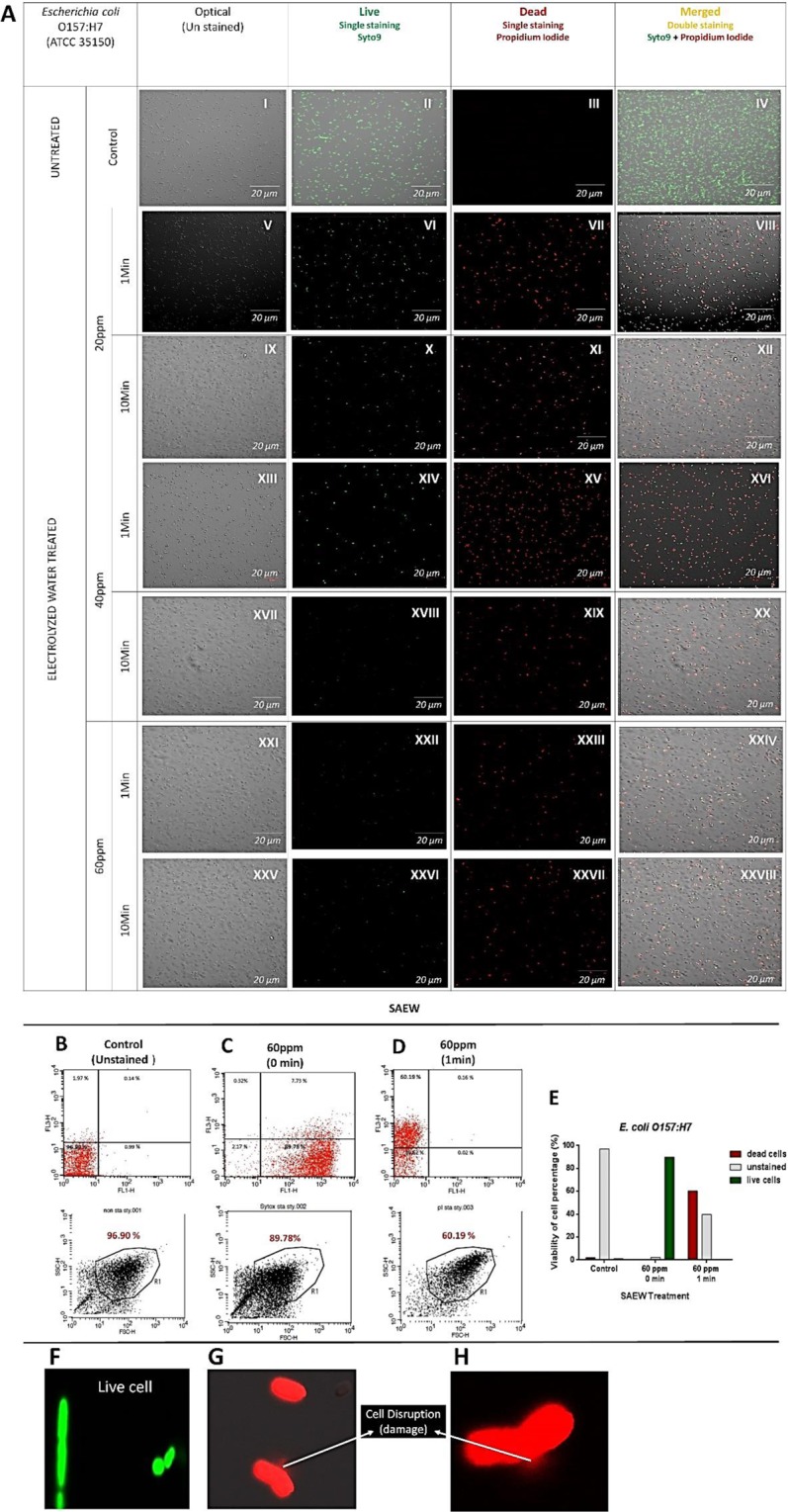
SR-CLSM imaging of *E. coli* O157:H7 bacterial cell death by LIVE/DEAD™ BacLight™ assay after treatment of Slightly Acidic Electrolyzed Water (20, 40, and 60 ppm) with different dipping times (1 and 10 mins) **(A)**. The live-dead proportion of *S.aureus* under Slightly Acidic Electrolyzed Water treatment (20 and 60 ppm) was assessed with a flow cytometry analysis; **(B)** Control (Unstained), **(C)** 60 ppm 0 min, **(D)** 60 ppm, 1 min. Live and dead cells percentage (%) under Slightly Acidic Electrolyzed Water (SAEW) treatment were represented as a chart **(E)**. SAEW treatment resulted in retaining *E, coli* morphological changes **(F–H)**. A live cell with no injury **(F)**, dead cells showing cell disruption **(G,H)**. Untreated control (AI-IV), SAEW 20 ppm treated for 1 min (AV-VIII), SAEW 20 ppm treated for 10 min (AIX- XII), SAEW 40 ppm treated for 1 min (AXIII- XVI), SAEW 40 ppm treated for 10 min (AXVII- XX), SAEW 60 ppm treated for 1 min (AXXI- XXIV), SAEW 60 ppm treated for 10 min (AXXV- XXVIII). Unstained bacterial cells (I, V, IX, XIII, XVII, XXI, XXV), Live cells – single syto9 staining (II, VI, X, XIV, XVIII, XXII, XXVI), Dead cells – single PI staining (III, VII, XI, XV, XIX, XXIII, XXVII), Merged cells – Double Syto9 + PI staining (IV, VIII, XII, XVI, XX, XXIV, XXVIII).

**Figure 4:**
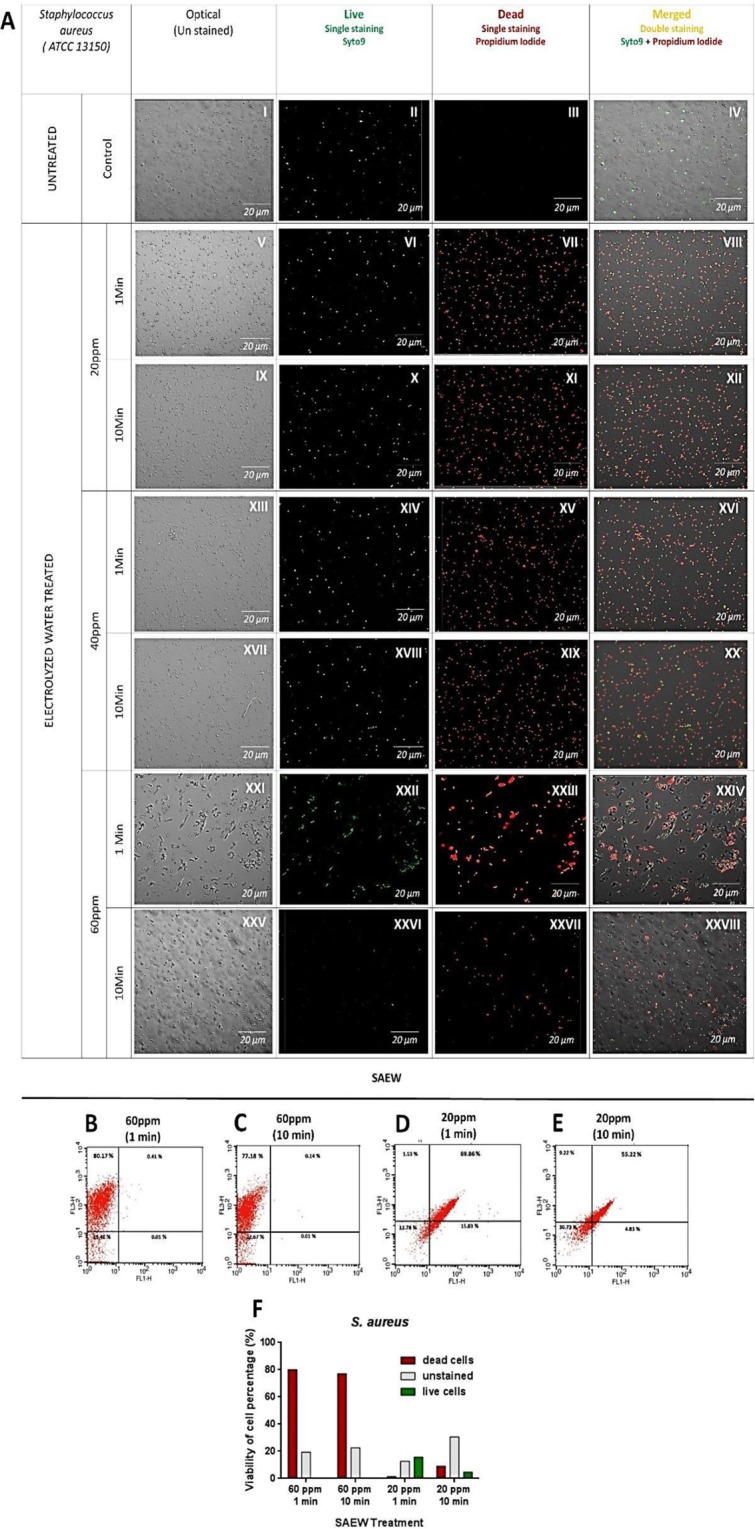
SR-CLSM imaging of *S. aureus* bacterial cell death by LIVE/DEAD™ BacLight™ assay after treatment of Slightly Acidic Electrolyzed Water (20, 40, and 60 ppm) with different dipping times (1 and 10 mins) **(A)**. The live-dead proportion of *S. aureus* under Slightly Acidic Electrolyzed Water treatment (20 and 60 ppm) was assessed with a flow cytometry analysis; **(B)** 60 ppm, 1 min, **(C)** 60 ppm, 10 min, **(D)** 20 ppm, 1 min, **(E)** 20 ppm, 10 min. Live and dead cells percentage (%) under Slightly Acidic Electrolyzed Water (SAEW) treatment were represented as a chart **(F)**. Untreated control (AI-IV), SAEW 20 ppm treated for 1 min (AV-VIII), SAEW 20 ppm treated for 10 min (AIX- XII), SAEW 40 ppm treated for 1 min (AXIII- XVI), SAEW 40 ppm treated for 10 min (AXVII- XX), SAEW 60 ppm treated for 1 min (AXXI- XXIV), SAEW 60 ppm treated for 10 min (AXXV- XXVIII). Unstained bacterial cells (I, V, IX, XIII, XVII, XXI, XXV), Live cells – single syto9 staining (II, VI, X, XIV, XVIII, XXII, XXVI), Dead cells – single PI staining (III, VII, XI, XV, XIX, XXIII, XXVII), Merged cells – Double Syto9 + PI staining (IV, VIII, XII, XVI, XX, XXIV, XXVIII).

SYT-PI Single and double staining of live and dead bacterial cells of *E. coli O157:H7* and *S. aureus* were used to prove the impact of SAEW on bacterial viability condition under a confocal laser-scanning microscopy **(**Figs 3A and 4A). Live cells were represented as green fluorescence **(**Fig. 3F**)** and dead cells were represented as red fluorescence (Fig. 3G,H).

Specifically 60 ppm of SAEW treatment showed more dead cells compared to 20 and 40 ppm. During the observation duration, 10 min of dipping time was considered to decrease the bacterial cell survival compared to 1 min of dipping time. It was observed that SAEW could penetrate the bacterial cell membrane at different concentrations (20, 40, and 60 ppm) along with cell damage and disruption. As shown in Fig. 3G,H, SAEW induced cell distention and led to cell disruption. The above results indicated that within 1 min of dipping, the bacterial cells were not completely inactivated; this data is quite incompatible with the results *in vitro* testing. This may be due to the different experiment procedures and method variations. The confocal data suggest that 60 ppm with 10 min of dipping would be influential to destroy bacterial cells.

### Flow cytometry Analysis

The change of the percentage of live and dead bacterial cells of *E. coli O157:H7* and *S. aureus* affected by SAEW was determined under flow cytometric analysis **(**Figs 3B–D and 4B–E). SYT-PI double staining was used to demonstrate the significance of SAEW on bacterial cells survival. When *E. coli* was treated with SAEW at 60 ppm with 1 min of dipping, the proportion of living cells were reduced rapidly (Fig. 3E). The percentage of dead cells increased to 60.19% indicating the bacterial inactivation under SAEW treatment (Fig. 3D). *S. aureus* was treated with SAEW at 20 and 60 ppm for comparison analysis. The viability rate of *S. aureus* cells maintained constant while treating with SAEW at 20 ppm regardless of dipping time (1 and 10 min) (Fig. 4D,E). However, 60 ppm of SAEW indicated that the high sanitizing potency in bacterial cells compared to 20 ppm (Fig. 4F).

### Transmission Electron Microscope (TEM) Analysis

**Figure 5:**
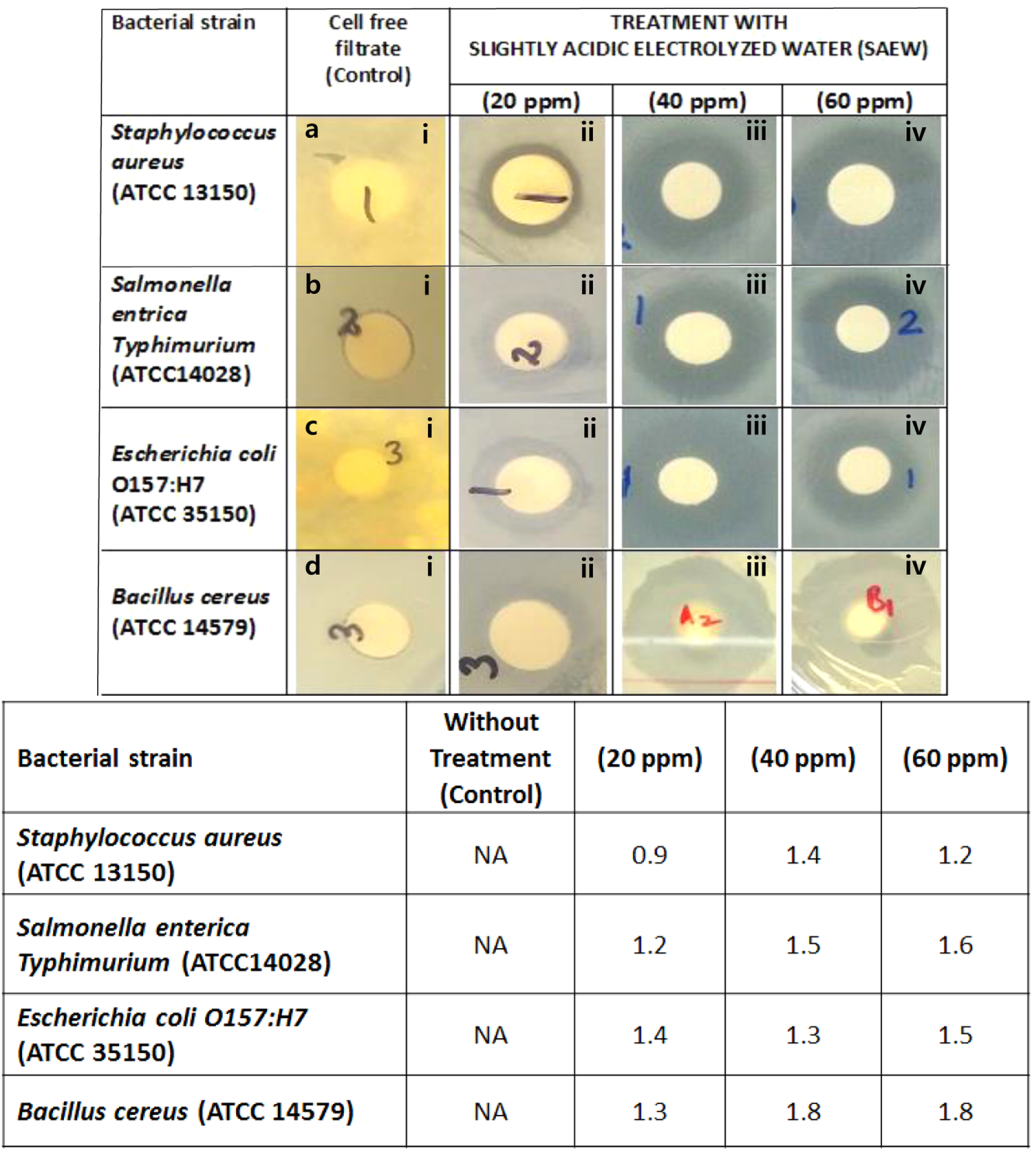
Transmission electron microscope indicating bacterial cell disruption based on the slightly acidic electrolyzed water (SAEW) treatment. Rigid cell wall of bacteria (i), cell wall damage (ii), cell inclusion breaches out of the cell (iii), thick wall bound spore (iv), cell wall damage at the posterior end (PE). *Salmonella enterica Typhimurium* (**A–D**), *Staphylococcus aureus* (**E–H**), *Bacillus cereus spores* (**I–L**), Untreated control bacterial cells under TEM (**A,E,I**), SAEW treated cells for 20 ppm (**B,F,J**), SAEW treated cells for 40 ppm (**C,G,K**), SAEW treated cells for 60 ppm (**D,H,L**), Entire cell shows disrupted (**D**).

Cell morphological state and cell permeability of *Salmonella enterica Typhimurium*, *S. aureus*, and *B. cereus* spores were observed through a transmission electron microscope and shown in Fig. 5. *S. enterica Typhimurium* and *S. aureus* bacterial cells were disrupted by SAEW treatment at 40 and 60 ppm and the cell wall membrane was damaged at the posterior end (Fig. 5C,D,G,H). In case of 20 ppm, the bacterial cells revealed partial minor damage in cell membrane **(**Fig. 5F**)**. These results indicated that treating SAEW at 60 ppm with 1 min led bacterial cell inclusion to be breached out of the cell. As shown in Fig. 5C,F, SAEW destroyed cell structure and the cell formation lost its usual and continuous shape. Due to the plasmolysis leaking from the intracellular components, cell fluidity and coherence were altered under the SAEW treatment^11^. However, *B. cereus* spores were unaffected by SAEW even at 60 ppm and remained as its rigid structure. There was no significant changes on the morphology of *B. cereus* spores occurred after SAEW treatment at 20, 40, and 60 ppm. These TEM pictures confirm that SAEW revealing the strong sanitizing efficacy lead to higher degree of cell membrane rupturing.

### Antimicrobial activity of slightly acidic electrolyzed water (SAEW) treatment against bacterial pathogens

**Figure 6:**
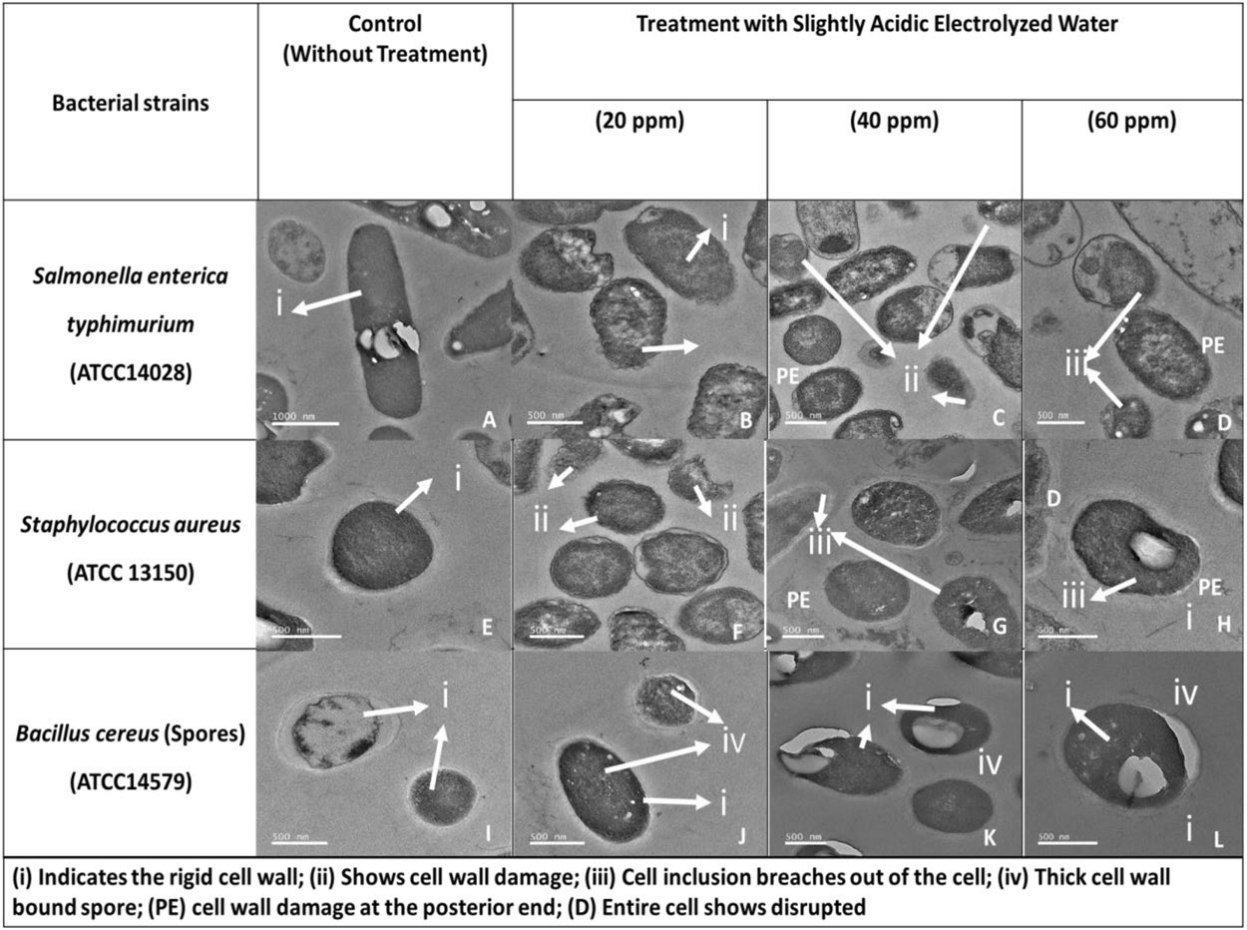
Antimicrobial activity of slightly acidic electrolyzed water (SAEW) treatment against bacterial pathogens. Staphylococcus aureus (**A** i–iv), Salmonella enterica Typhimurium (**B** i–iv), Escherichia coli O157:H7 (**C** i–iv), Bacillus cereus (**D** i–iv). Untreated control bacterial cells (ai,bi,ci,di), Treated with 20 ppm SAEW (aii,bii,cii,dii), Treated with 40 ppm SAEW (aiii, biii, ciii, diii), Treated with 60 ppm SAEW (aiv, biv, civ, div).

Antimicrobial properties of slightly acidic electrolyzed water (SAEW) treatment was evaluated against Gram positive and Gram negative bacterial pathogens and the results were shown in Fig. 6. The results revealed that different concentrations (20, 40, and 60 ppm) of SAEW showed the antimicrobial activity. Among the SAEWs, SAEW containing 60 ppm was the most effective retarding microbial growth of pathogens. Distilled water (without treatment) did not show the antimicrobial activity. SAEW containing 20, 40, and 60 ppm represented 0.9 to 1.2 mm zone of inhibition against *S. aureus* (ATCC 13150) and 1.2 to 1.6 mm zone of inhibition against *S. enterica Typhimurium* (ATCC 14028). In addition, SAEW retaining 20, 40, and 60 ppm indicated 1.3 to 1.5 mm of inhibition zone for *E. coli* O157:H7 (ATCC 35150) and 1.3 to 1.8 mm for *B. cereus* (ATCC 14579).

The results show that SAEW can completely inactivate *E. coli*, *S. aureus*, *S. enterica*, and *B. cereus* at 60 ppm water hardness or higher within 10 min of treatment (Figs 2, 5 and 6). However, within 1 min, the population of *E. coli* O157:H7 (ATCC 35150) was decreased only 3.90 and 3.77 log, respectively, with complete inactivation after 10 min only in SAEW. However, the population of *E. coli* O157:H7 was significantly lower after 2 min compared to 1 min (P < 0.05). Results showed that *E. coli* O157:H7 was more resistant to SAEW and within 30 sec, reductions were not observed respectively. Little is known about the emerging foodborne pathogen *E. coli* O157:H7, especially how to governor it. Therefore, these results are important for sterilize of water, produce and fresh produce. Former treatments for worthy manufacturing and sanitation practices, as well as those set forth by HACCP (Hazard Analysis Critical Control Point) programs, were inadequate for incapacitating this harmful pathogen.

The levels of water hardness significantly affected the efficacy of EO water in activating *S. enterica* and *S. aureus* (Fig. 5). The Overall, increasing water hardness from (34 ± 2 mg/L) 20ppm to 60 ppm (55–60 mg/L) significantly increased the reduction of *S. enterica* and *S. aureus* from 1.31 log CFU/mL after treatment of 1.0 ml culture in 9.0 ml of EO water. This increased reduction was in line with changes in hardness based EO water properties. Improbably, further increase of water hardness to below 20ppm had no significant effect on the overall reduction of the pathogen, although this hardness increase significantly raised Available Chlorine Concentrations levels (ACC) of EO water (Figs 2, 5 and 6).

## Discussions

Due to the industrialization and growing population, the water resources have been problematic nowadays. Agricultural pollutants, such as chemical fertilizers, pesticides, and industrial disposals have been soaked inside the soil and wells and debased the water quality. This may degrade the mineral contents in groundwater and lead to lower water hardness^10^.

Increase of water hardness might have augmented electrolyte concentration, and conductivity or electrical current in the electrolytic solutions, therefore, more free chlorine would be obtained. These results indicate that different possible water hardness should be taken into consideration while planning a sanitization approach for a food plant /facility or acquiring EW generators. Similar results were observed by Pangloli and Hung^12^ that higher water hardness led to increased available chlorine concentration (ACC), oxidation-reduction potential (ORP), and decreased pH of EW.

There are many factors affecting on the properties of EW, such as water temperature, electrolyte flow rate, salt concentration, and electrode materials^5,13^. Aside from them, water hardness must also be concerned as the crucial factor for SAEW production when optimizing the process. Due to the difference in water hardness, some waters would have less potential to produce proper SAEW and it will be in need of optimizing the production conditions by changing amperage, electrolyte concentrations, or in addition of salts^14^.

The combination of HCL and NaCL showed higher ACC and lower pH values than those observed in combination of HCL and KCL. This difference may appear due to the high NaCl concentration used herein. With high NaCl and free chlorine concentrations, strong acidic EW had higher germicidal effect than that of strong acidic EW with low NaCl and free chlorine concentrations^15^.

The optimization of SAEW demands a better combination of these factors. The use of KCL can be preferred to NaCl. The results showed three more SAEW production (total 9 SAEWs) in the combination of HCL + KCL compared to those in the combination of HCL + NaCl (total 6 SAEWs). When SAEW is applied to plant or plant components, potassium has a beneficial activity increasing cell osmotic pressure and stress tolerance, while sodium is capable to induce leaf edge dehydration and necrosis^16^.

In the present study, the addition of 5% HCL + 2.0 M NaCl at 1.50 mL/min flow rate was considered to be the finest electrolyte concentration for optimizing SAEW production from water hardness of 34 ± 2 mg/L and obtained ACC of 29 ppm^17^. On the other hand, our results showed the addition of 6% HCL + 3.0 M NaCl at 1.00 mL/min EFR was determined to be the best electrolyte condition using water hardness of 29 mg/L and gained ACC of 40.5 ppm, which is much higher than the one in the previous study.

The SAEW reduction resulted from previous studies reported 2 min was enough to completely inactivate *B. cereus* spores by SAEW with ACC of 55–60 mg/L^18^. However, our results showed bacterial reduction of 1.31 log CFU/mL after 1 min under SAEW (pH 5.0, ACC 20 ppm). These results were almost in agreement with the results from Kim and others^15^ that increasing treatment time to 2 min caused spores reduction by electrolyzed oxidizing water to 1.4 log CFU/mL. *B. cereus* spores were more resistant to the treatments than vegetative cells. When *B. cereus* spores went through striking physical metabolic adaptations in return to harsh and stagnant environmental circumstances, they led to sporulation time. During cryptobiosis, spores suffered and could possibly cause dormant food spoilage^19,20^. Spores inactivation may be affected by variation between strains and experimental conditions^21^. Moreover, the results could vary depending on the medium and how the spores were prepared^22^.

In addition, after 30 s of dipping*, B. cereus* vegetative cells were observed to be completely inactivated under EO water (pH 2.6, ORP 1160 mV, Cl 56 mg/L)^15^, while our results indicated *B. cereus* vegetative cells to become complete inactivation within 1 min of dipping under 20 ppm of SAEW.

Fluorescent lights from PI and Syto-9 estimated viable and dead cells. Syto-9 stain marked all bacteria, which were those with complete cell membranes and those with injured membranes. Since PI had too low intensity compared to Syto-9, its attraction to target nucleic acids became much greater than Syto-9. Therefore, PI penetrated only bacteria with spoiled membranes, leading to a decrease in the Syto-9 fluorescent stain. As a result, PI intercalated between base pairs and bound to double stranded DNA through damaged or ruptured cells membranes^23^.

For germicidal process, morphological transformations were often caused by disinfectants or sanitizers^24^. We detected that *E. coli* was disrupted and injured by SAEW (Fig. 3G,H). SAEW could have a decisive effect on the *E. coli* morphology. Such morphological changes were observed from previous studies that electrolyzed oxidizing water fortified membrane permeability and the conductivity in bacterial suspension was enhanced, resulting in a release of K^+^ and protein out of *Bacillus subtilis* cells^25^. However, in case of *B. cereus* spores still maintained its rigid cell membranes without splitting or damage following SAEW treatment.

The flow cytometric results indicated that SAEW generated apoptosis features in bacterial cells (*S. aureus* and *E. coli*). SAEW may as well harm bacterial cells than killing it promptly. These data are similar to the inference of the study reported by Ye, Z. *et al*.^12^.

As per the previous studies conducted by Hsu^14^ studied the effects of water flow rate, salt concentration, and water temperature on pH, oxidation-reduction potential (ORP), total residual chlorine, dissolved oxygen, electrical conductivity, and salinity of EW. Increasing salt concentration elevated total chlorine concentration and electrical conductivity of EW. They also found out that ORP decreased with increases in water flow rate. This result was different from our studies that increasing flow rate resulted in increasing ORP in our studies.

Hsu^10^ also investigated the effects of water flow rate, water temperature, and salt concentration on electrolysis efficiency and separation efficiency of EW generator, operating different electric potential (7.9–15.6 V) and power consumption (16–120 W) of the electrolysis cell. Electrolysis efficiency of the electrolysis cell varied in the range of 23–51% and electric current of the cells differed depending on water flow rate and water temperature.

Jeong *et al*.^26^ carried out experiments to study the efficacy of surface sterilization and the physicochemical properties of EW manufactured from various electrolytic diaphragm and electrolyte. The most effective diaphragm system was that the distance between diaphragm 1.0 mm, and supplying rate of 20% NaCl was 6 mL/min. At that moment, the pH, ORP, and HOCl content were 2.5, 1,170 mV, and 100 ppm, respectively. In our study, the optimal condition to produce the finest EW was electrolyte flow rate of 2 mL/min, electrolyte as 4% HCL + 3.0 M KCL. The pH, ORP, and HOCl content at above condition were 5.66, 800–889 mV, and 65.5 ppm.

Seo^27^ treated with SAEW (pH 2.3 and a chlorine concentration of 49 ppm) for 10 mins against *E. faecalis* biofilms. After treating with SAEW, the green fluorescence intensity decreased, and the red fluorescence intensity increased on both the flow and static *E. faecalis* biofilms. SAEW showed significantly greater bacterial reductions of 88.2% for the flow biofilms and 90.3% for the static biofilms. In our study, SAEW of 40 ppm within 10 min of dipping time was used to find out the bacterial viability against *E. coli* O157:H7 and S. aureus. The results revealed the increased red fluorescence intensity and reduced green florescence (Sytox green).

During the bactericidal process, bacterial structural changes often can be persuaded by antibiotics or sanitizers^28^. Here, we establish that the *S. entrica typhimurium*, *S. aureus*, *Bacillus cereus* spores elongated and were bloated by SAEW. SAEW at 20ppm could change the *S. entrica typhimurium* and *S. aureus*, morphology but still maintain their cell shape, which indicated that the cells may not disrupt or spilt following SAEW treatment. However, the cell shape existence does not imply the cellular function still existence. We found the cell permeability increased at 40–60 ppm, which means the cell membrane integrity was destroyed. In terms of changes of cell membrane permeability, our postulation was verified when further the Propidium iodide (PI) fluorescence increased following SAEW treatment. These phenomena can also be observed with different stimuli, such as heat and high pressure sterilization on bacteria with irreversible loss of membrane integrity, as indicated by PI uptake^29,30^. Wenwei Tang *et al*.^25^, hence it is found that electrochemical oxidizing water (EOW) could strengthen membrane permeability, improve the conductivity of suspension and cause leakage of cell inclusion out of Bacillus vegetative cells. They found that the cell wall and membrane were broken, which delivers an impending description for how SAEW may act. But the *Bacillus* spores were found intact during SAEW treatment.

## Conclusion

Based on the study, it was concluded that hardness of starting water is the significant factor of SAEW production process and has to be taken into consideration. TW having water hardness of 29 ppm has more potential to produce better SAEW than UGW of 12 ppm. Low water hardness can be reinforced by adding the combination of hydrochloric acid with salts. The electrolyte combination of HCL and KCL revealed more SAEW formation than those of HCL and NaCl. SAEW manifested a convincing sanitizing effect on foodborne pathogens through cell viability investigations. Further studies should be continued and applications to food samples *in vivo* will be necessary. Finding different electrolytes would be of great importance for further studies.

## Materials and Methods

### Bacterial strains preparation

Two strains of *Escherichia coli* O157:H7 (ATCC 35150, 496), *Staphylococcus aureus* (ATCC 13150, 12600), *Salmonella enterica* (ATCC 14028, 13076)*, and Bacillus cereus* (ATCC 14579, 10987) were used in this study. Strains were individually transferred into 10 mL of tryptic soy broth (TSB; Difco, Sparks, MD, USA) and incubated for 24 h at 37 °C. Each culture was collected as pellet using centrifugation (4000 × g for 10 min at 4 °C), washed twice in 10 mL of 0.1% buffered peptone water (BPW, Difco). A cocktail of each pathogen was prepared by mixing 10 mL of each strain. Cocktail contains approximately 8–9 log CFU/mL. Cocktail population was determined by plating 0.1 mL of each serial dilution into tryptic soy agar (TSA, Difco) and incubating at 37 °C for 24 h. *B. cereus* spores were prepared using the method described by Dufrenne *et al*.^31^ Spore population was 6–7 log CFU/mL and was checked as abovementioned.

### Water sources and SAEW preparation

Two different types of water were used to produce EW under the same conditions: TW from KNU, Chuncheon-Si, Kangwon-do, South Korea. UW from Jongja-ri Rd, Dongsan-myeon, Chuncheon-si, Kangwon-do, South Korea. The hardness of both waters was measured by Department of drinking water analysis, Institute of Health and Environment, Chuncheon-Si, Kangwon-do, South Korea.

Firstly, EW was prepared using a self-developed electrolysis generator without membrane between anode (IrO2 + SnO2) and cathode (Ti) as shown in Fig. 1 and Supplement 1, 2^32^. Water from different sources was mixed inside of electrolytic cell with 6.0% HCL and the cell was run at 8A. Water recirculation rate was adjusted by means of a valve to 2 L/min. HCL concentration and amperage were selected based on our previous studies^7^. The pH, ORP, and ACC of SAEW were measured with a dual scale pH meter (Accumet model 15, Fisher Scientific Co., Fair Lawn, NJ, USA) bearing pH and ORP electrodes. ACC was determined by a colorimetric method using a digital chlorine test kit (RC-3F, Kasahara Chemical Instruments Corp., Saitama, Japan).

### Optimization of SAEW production

TW and UW were used for the optimization of SAEW production by using modified procedure of Naim *et al*.^33^. Water flow rate remained constant at 2 L/min and various salt concentrations, current, and electrolyte flow rate (EFR) were used in order to discover the optimal condition which can allow the production of SAEW with high ACC value, while the setting of current was fixed at 12.0A. A 500 mL flask was used for combining different concentration of NaCl and KCL with 4 and 6% HCL, respectively. The SAEW properties were measured as abovementioned.

### Sanitization efficacy of SAEW against pathogens

Effect of different ACCs (20, 40 and 60 ppm) and pH ranges (5.0, 5.5, 6.0, and 6.5) of SAEW was investigated against pure cultures of *E. coli* O157:H7, *S. aureus*, *Salmonella spp., B. cereus* vegetative cells, and *B. cereus* spores at room temperature (23 ± 0.2 °C) for different dipping times (1, 3, 5, and 10 min).

*In vitro* inactivation of pathogens was performed using the method by Issa-zacharia *et al*.^2^ as shown in Supplement 1. Two replicates were performed in duplicate and bacterial populations were expressed as log CFU/mL. The detection limit of the method was 1 log CFU/mL.

### Confocal laser scanning microscopy analysis

The treated (SAEW) and untreated bacterial cell were centrifuged (4000 × g for 15 min at 4 °C) and suspended in 0.1% buffered peptone water^11^. The morphological changes, dead and live cells of bacterial cells were documented using super sensitive high resolution confocal laser scanning microscope imaging (SR-CLSM; LSM880 with Airyscan, ZEISS, Oberkochen, Germany) and Live/dead cells were stained with Syto-9 (SYT) and Propidium Iodide (PI) respectively; Propidium Iodide (Laser Line-488nm; Excitation-535; Emission-488) and Syto-9 (Laser Line- 488 nm; Excitation -617;Emission- 503).

### Flow cytometry analysis

The treated (SAEW) and untreated bacterial cell were centrifuged (4000 × g for 15 min at 4 °C) and suspended in 0.1% buffered peptone water^12^. Based on Syto-9 (SYT) and Propidium Iodide (PI), live and dead cells were stained respectively; Propidium Iodide (Laser Line-488nm; Excitation-535; Emission-488) and Syto-9 (Laser Line- 488 nm; Excitation -617; Emission- 503). The percentage rate of live and dead cells was determined. The treated samples were observed with a FACS Calibur flow cytometer (Benton Dickinson, USA) to obtain data using the bandpass filter (670 LP, 530/30).

### Transmission electron microscope (TEM) analysis

TEM technique was used to observe cell disruption images^12^. The treated (SAEW) and untreated bacterial cell were centrifuged (4000 × g for 15 min at 4 °C) and suspended in 0.1% buffered peptone water. The bacterial cells were then fixed in 4% glutaldehyde and 1% paraformaldehyde solution in 0.1 M cacodylate buffer (pH 7.4) for 3~4 hours. 4% glutaldehyde solution was pipetted off and the samples were rinsed in 0.1 M cacodylate buffer (pH 7.4) three times for 10 minutes. After treating with ethanol, propylene oxide, and Eponate 812 resin, the samples were baked at 65 °C for 24 hours. The bacterial cells were sectioned using Ultra microtome. The morphological changes were observed under Energy-Filtering Transmission Electronic Microscope (EF-TEM, LEO912AB, Carl Zeiss).

### Antimicrobial activity assay

Each bacterial strains were prepared in selective broth at 37 °C with 150 RPM for 16–18 hours. The bacterial growth was harvested using 0.1% buffered peptone water, its absorbance was adjusted to 600 nm and diluted to reach viable cell count of 10^8^ CFU.mL^−1^ using spectrophotometer. The disc diffusion method was adapted to assess the antimicrobial activity of slightly acidic electrolyzed water (SAEW) treatment. Each microbial suspension (100 μL) was inoculated onto the media using spreader. Autoclaved filter paper disc (8 mm in diameter) loaded with different concentrations of SAEW (20, 40, and 60 ppm) were aseptically placed on the top of agar surface. The inoculated plates were allowed to stand at room temperature for 30–45 min to allow diffusion of SAEW prior to incubation at 37 °C for Gram positive and Gram negative bacteria. At the end of incubation, inhibition zones formed around the disc were measured with transparent ruler in millimeter^12^.

### Data processing

Means of bacterial populations (log cfu/mL) from each treatment were subjected to analysis of variance (ANOVA) using IBM SPSS Statistics Version 21 (SPSS Inc., An IBM Company). Tukey’s multiple range test was used with the significance of difference defined at p < 0.05^7^.

### Data Citations

1. Schematic diagram of the electrolyzed water generator system.
https://figshare.com/s/3e7a023af322ef1505eb
2. A schematic mechanism illustration of the slightly electrolyzed water generator system.
https://figshare.com/s/bc13aae77ab66a05f499
3. Sanitizing experiments process using SAEW and sodium thiosulphate solution.
https://figshare.com/s/26ea55fe002c1655d6e7
4. Properties of different waters used in EW production.
https://figshare.com/s/1c9475b5382601cfe111
5. EW properties produced from TW and UW starting waters using various electrolyte flow rate and fixed electrolyte concentration (6% HCl) and amperage.
https://figshare.com/s/3cc697f23138fe0f221c
6. Optimization of Tap and underground water SAEW production system by combining 1.0–3.0 M KCl with 4% HCl and its physicochemical properties.
https://figshare.com/s/e2eb8db4685254158c3c
7. Optimization of Tap and underground water SAEW production system by combining 1.0–3.0 M NaCl with 6% HCl and its physicochemical properties.
https://figshare.com/s/4d1a18ff2268dad426bb
8. Effect of SAEW concentrations and different pH on the inactivation of pathogens. Effect of SAEW concentrations and different pH in combination with dipping times on SAEW sanitization efficacy against *B. cereus* spores.
https://figshare.com/s/24cb8fc6ce2e3c423d1a
9. Viability observations of *E. coli* O157:H7 by differential/double staining of living and dead cells after SAEW treatment under a confocal microscope **(A)**. The live-dead percentage of *E. coli* under SAEW treatment (60 ppm) was assessed with a flow cytometry at the following: **(B)** Control (Unstained), **(C)** Live cells **(D)** Dead cells. Live and dead cells percentage (%) under SAEW treatment were represented as a chart **(E)**. SAEW treatment resulted in retaining *E,coli* morphological changes. **(F)** a Live cell with no injury **(G), (H)**, Dead cells showing cell damage.
https://figshare.com/s/f62556e125c4c1ec3b23
10. Viability observations of *S.aureus* by differential/double staining of living and dead cells after SAEW treatment under a confocal microscope **(A)**. The live-dead percentage of *S.aureus* under SAEW treatment (20 and 60 ppm) was assessed with a flow cytometry at the following: **(B)** 60 ppm, 1 min, **(C)** 60 ppm, 10 min, **(D)** 20 ppm, 1 min, **(E)** 20 ppm, 10 min. Live and dead cells percentage (%) under SAEW treatment were represented as a chart **(F)**.
https://figshare.com/s/be2497f6c4f2d3331663
11. Transmission electron microscope indicating bacterial cell disruption based on the slightly acidic electrolyzed water (SAEW) treatment.
https://figshare.com/s/7d2cee540e59122e6911
12. Antimicrobial activity of slightly acidic electrolyzed water (SAEW) treatment against bacterial pathogens.

https://figshare.com/s/a6dabac94c32e5e158af

## Supplementary information

Supplement Information
