# Supplementary material for: Sanitization Efficacy of Slightly Acidic Electrolyzed Water against pure cultures of Escherichia coli, Salmonella enterica, Typhimurium, Staphylococcus aureus and Bacillus cereus spores, in Comparison with Different Water Hardness
Source: Sci Rep. 2019 Mar 13;9:4348. doi: 10.1038/s41598-019-40846-6 (PMC6416306; doi:10.1038/s41598-019-40846-6)
Supplement: Supplementary file 1 — Supplement Information [file 41598_2019_40846_MOESM1_ESM.pdf]

Sanitization Efficacy of Slightly Acidic Electrolyzed Water against pure cultures of *Escherichia coli, Salmonella enterica, Typhimurium, Staphylococcus aureus and Bacillus cereus* spores, in Comparison with Different Water Hardness

Hyun-Ji Kim[1], Charles Nkufi Tango[1], Ramachandran Chelliah[1]*, Deog-Hwan Oh*

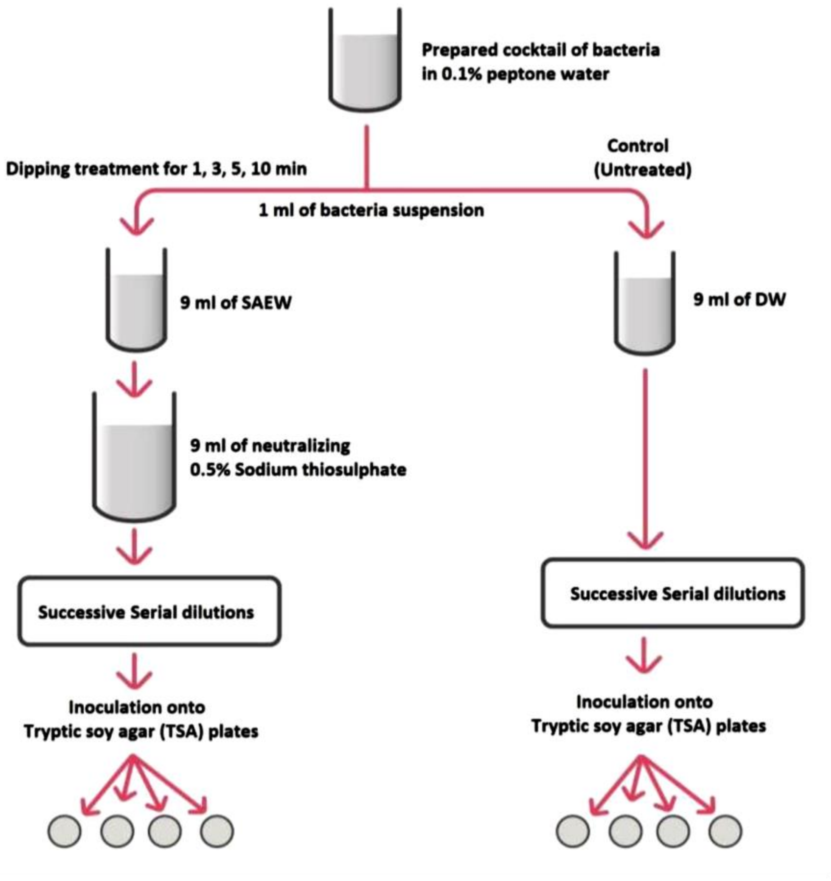

Supplement 1. Flow diagram showing experiments designed to determine sanitization potency of SAEW on pure cultures of foodborne pathogens treated for 1, 3, 5, 10 min. Sodium thiosulphate solution (0.5%) was used to stop the inactivation reaction.

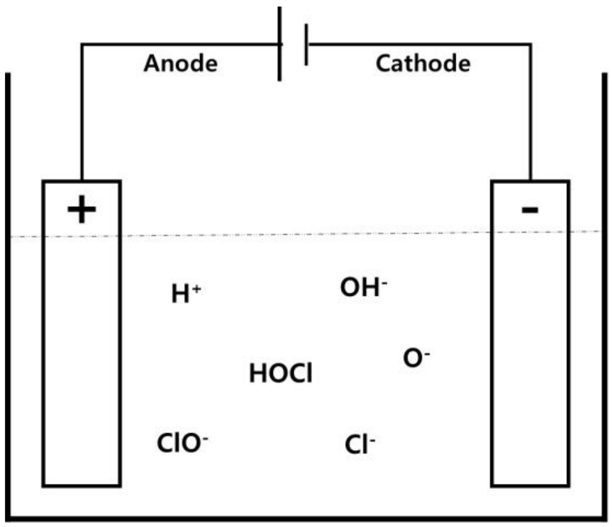

Supplement 2. A schematic mechanism illustration of the slightly electrolyzed water generator system

A - Tap Water

#	Inspection items	Water Quality Standards	Inspection Results	
			Before Production	After Production
1	Total colony Counts	100CFU/mℓ or less	0	0
2	Total Coliforms	ND/100mℓ or less	ND	ND
3	Fecal Coliforms	ND/100mℓ or less	ND	ND
4	Pb; Lead	0.01mg/ℓ or less	ND	ND
5	F; Fluoride	1.5mg/ℓ or less	0.19	0.21
6	As; Arsenic	0.01mg/ℓ or less	ND	ND
7	Se; Selenium	0.01mg/ℓ or less	ND	ND
8	Hg; Mercury	0.001mg/ℓ or less	ND	ND
9	CN; Cyanide	0.01mg/ℓ or less	ND	ND
10	Cr^{+6}; Hexachromium	0.05mg/ℓ or less	ND	ND
11	NH_3-N; Ammonium Nitrogen	0.5mg/ℓ or less	ND	ND
12	NO3-N; Nitrate Nitrogen	10mg/ℓ or less	1.1	2.1
13	Cd; Cadmium	0.005mg/ℓ or less	ND	ND
14	B; Boron	0.3mg/ℓ or less	ND	ND
15	Phenol	0.005mg/ℓ or less	ND	ND
16	Diazinon	0.02mg/ℓ or less	ND	ND
17	Parathion	0.06mg/ℓ or less	ND	ND
18	Fenitrothion	0.04mg/ℓ or less	ND	ND
19	Carbaryl	0.07mg/ℓ or less	ND	ND
20	1.1.1-Trichloroethane	0.1mg/ℓ or less	ND	ND
21	PCE; Tetrachloroethylene	0.01mg/ℓ or less	ND	ND
22	TCE; Trichloroethylene	0.03mg/ℓ or less	ND	ND
23	1.1 Dichloroethylene	0.03mg/ℓ or less	ND	ND

24	Dichloromethane	0.02mg/ℓ or less	ND	ND
25	Benzene	0.01mg/ℓ or less	ND	ND
26	Ethyle Benzene	0.3mg/ℓ or less	ND	ND
27	Carbon tetrachloride	0.002mg/ℓ or less	0.001	0.001
28	Xylene	0.5mg/ℓ or less	0.001	0.12
29	Toluene	0.7mg/ℓ or less	0.003	0.001
30	1,2-Dibromo-3-Chloropropan	0.003mg/ℓ or less	ND	ND
31	Hardness	300mg/ℓ or less	29	82
32	Consumption of KMnO4	10mg/ℓ or less	0.5	3
33	Odor	ND	ND	ND
34	Taste	ND	ND	ND
35	Cu; Cooper	1mg/ℓ or less	0.015	0.018
36	Color	5 or less	1	3
37	ABS;<br>Alkyl Benzene Sulfate	0.5mg/ℓ or less	ND	0.1
38	pH	5.8 ~ 8.5	6.86	5.66
39	Zn; Zinc	3mg/ℓ or less	0.012	0.011
40	Cl-; Chloride	250mg/ℓ or less	8	13
41	Total Solds	500mg/ℓ or less	142	138
42	Fe; Iron	0.3mg/ℓ or less	ND	ND
43	Mn; Manganese	0.3mg/ℓ or less	ND	ND
44	Turbidity	0.5 NTU or less	0.14	0.23
45	SO4-2; Sulfate	200mg/ℓ or less	5	5
46	Al; Aluminium	0.2mg/ℓ or less	ND	ND

B - Underground Water

#	Inspection items	Water Quality Standards	Inspection Results	
			Before Production	After Production
1	Total colony Counts	100CFU/㎖ or less	0	0
2	Total Coliforms	ND/100㎖ or less	ND	ND
3	Fecal Coliforms	ND/100㎖ or less	ND	ND
4	Pb; Lead	0.01㎎/ℓ or less	ND	ND
5	F; Fluoride	1.5㎎/ℓ or less	0.28	0.26
6	As; Arsenic	0.01㎎/ℓ or less	ND	ND
7	Se; Selenium	0.01㎎/ℓ or less	ND	ND
8	Hg; Mercury	0.001㎎/ℓ or less	ND	ND
9	CN; Cyanide	0.01㎎/ℓ or less	ND	ND
10	Cr^{+6}; Hexachromium	0.05㎎/ℓ or less	0.01	0.02
11	NH_3-N; Ammonium Nitrogen	0.5㎎/ℓ or less	ND	ND
12	NO3-N; Nitrate Nitrogen	10㎎/ℓ or less	3.1	3.5
13	Cd; Cadmium	0.005㎎/ℓ or less	ND	ND
14	B; Boron	0.3㎎/ℓ or less	ND	ND
15	Phenol	0.005㎎/ℓ or less	ND	ND
16	Diazinon	0.02㎎/ℓ or less	ND	ND
17	Parathion	0.06㎎/ℓ or less	ND	ND
18	Fenitrothion	0.04㎎/ℓ or less	ND	ND
19	Carbaryl	0.07㎎/ℓ or less	0.01	0.01
20	1.1.1-Trichloroethane	0.1㎎/ℓ or less	ND	ND
21	PCE; Tetrachloroethylene	0.01㎎/ℓ or less	ND	ND
22	TCE; Trichloroethylene	0.03㎎/ℓ or less	ND	ND
23	1.1 Dichloroethylene	0.03㎎/ℓ or less	ND	ND

24	Dichloromethane	0.02mg/ℓ or less	ND	ND
25	Benzene	0.01mg/ℓ or less	ND	ND
26	Ethyle Benzene	0.3mg/ℓ or less	ND	ND
27	Carbon tetrachloride	0.002mg/ℓ or less	0.001	0.001
28	Xylene	0.5mg/ℓ or less	ND	ND
29	Toluene	0.7mg/ℓ or less	0.008	0.01
30	1,2-Dibromo-3-Chloropropan	0.003mg/ℓ or less	ND	0.001
31	Hardness	300mg/ℓ or less	12	66
32	Consumption of KMnO4	10mg/ℓ or less	2	5
33	Odor	ND	ND	ND
34	Taste	ND	ND	ND
35	Cu; Cooper	1mg/ℓ or less	0.2	0.25
36	Color	5 or less	1	2
37	ABS; Alkyl Benzene Sulfate	0.5mg/ℓ or less	ND	ND
38	pH	5.8 ~ 8.5	6.97	5.93
39	Zn; Zinc	3mg/ℓ or less	0.02	0.016
40	Cl-; Chloride	250mg/ℓ or less	0.2	0.76
41	Total Solds	500mg/ℓ or less	121	146
42	Fe; Iron	0.3mg/ℓ or less	0.003	0.009
43	Mn; Manganese	0.3mg/ℓ or less	ND	ND
44	Turbidity	0.5 NTU or less	0.2	0.56
45	SO4-2; Sulfate	200mg/ℓ or less	15	17
46	Al; Aluminium	0.2mg/ℓ or less	ND	ND

Supplement 3. Water Quality Analysis Report of Tap Water Before and After EW production **(A)** and Underground water before and after EW production **(B).**

* Water Quality Analysis has been completed by a drinking water quality testing agency, the Korean Institute of Environmental Hygiene, Yangpyeong-gun, Gyeonggi-do, South Korea.
